# From Test Offer to Urologic Follow-up: Acceptability of Semen Analysis in Male Infertility and Preconception Care

**DOI:** 10.7759/cureus.114929

**Published:** 2026-08-21

**Authors:** Kosuke Kojo, Ayumi Nakazono, Tomoko Oguri

**Affiliations:** 1 Department of Urology, University of Tsukuba Hospital, Tsukuba, JPN; 2 Tsukuba Clinical Research &amp; Development Organization (T-CReDO), University of Tsukuba Hospital, Tsukuba, JPN; 3 Center for Human Reproduction, International University of Health and Welfare Nasu Medical Center, Nasushiobara, JPN; 4 Research Institute of Science for Safety and Sustainability, National Institute of Advanced Industrial Science and Technology (AIST), Tsukuba, JPN

**Keywords:** acceptability, male infertility, men’s health, preconception care, semen analysis, service design

## Abstract

Semen analysis is a basic component of male infertility evaluation and can serve as an entry point to male reproductive health and couple-based fertility care. However, its acceptability is shaped by features that differ from many routine laboratory tests, including the private and sexual nature of specimen collection, abstinence and timing requirements, privacy concerns, result anxiety, stigma, and uncertainty about clinical follow-up.

This targeted narrative review synthesizes clinical, behavioral, psychosocial, technological, and Japanese-context evidence relevant to semen-analysis acceptability in male infertility and preconception/couple care. Rather than proposing or validating a new acceptability model, it presents an author-developed, provisional nine-stage map of semen-analysis service events: information reach, comprehension, self-relevance, emotional safety, collection feasibility, informed decision, sample submission, result interpretation, and care connection. It uses established acceptability and health-behavior concepts to interpret where attrition may occur. The map is conceptual and hypothesis-generating; its stages, boundaries, and candidate measures require prospective operationalization and validation.

This pathway reframes low uptake or noncompletion as possible attrition before, during, or after testing, rather than as simple refusal. Evidence suggests that barriers may include limited fertility knowledge, fertility being framed as a women’s issue, embarrassment, fear of abnormal results, stigma, collection logistics, and unsupported result return. Potential stage-specific responses include private and plain-language communication, non-shaming framing, feasible collection options, contextual result explanation, repeat-testing guidance, and referral to urology or male infertility care when indicated.

Home collection and home-based or point-of-care tools may reduce procedural barriers, but they should not become unsupported endpoints without interpretation and care linkage. Improving semen-analysis acceptability should support informed, voluntary, unashamed, and clinically connected decision-making within equitable couple care.

## Introduction and background

Why semen-analysis acceptability matters

Semen analysis is a core part of infertility evaluation [1-3]. It provides laboratory information on semen volume, sperm concentration, motility, morphology, and related parameters according to standardized methods [4,5]. The sixth edition of the World Health Organization (WHO) Laboratory Manual provides standardized procedures and quality-control principles for laboratory examination and reporting, thereby supporting the quality and comparability of results across laboratories [4,5]. It is a laboratory reference rather than a clinical practice guideline for treatment or referral decisions. Japanese male infertility guidance places semen evaluation within a broader urological pathway, while the current American Urological Association (AUA) and the American Society for Reproductive Medicine (ASRM) guideline, as amended in 2024, frames male evaluation as part of the concurrent assessment of an infertile couple [1-3]. Thus, semen analysis is a practical entry point into male reproductive evaluation and should not be treated only as a supplementary test after female evaluation has already advanced.

The reason to make semen analysis acceptable is broader than the chance of conception from one test result. Male reproductive function has been discussed as a possible window into broader men’s health, including cardiometabolic, endocrine, genetic, oncologic, and lifestyle-related conditions [6]. Longitudinal evidence also suggests that semen quality may be associated with later morbidity [7]. Still, semen analysis should not be used as a stand-alone general health screening test. A safer clinical interpretation is that abnormal or persistent semen findings can provide an opportunity for appropriate male reproductive and general health assessment, if the findings are clearly explained and connected to care.

Clinical importance alone does not mean that men can or will complete the test. Semen analysis differs from many other laboratory tests. It requires production of a private sexual specimen, usually by masturbation, under constraints of place, timing, and privacy, and may yield a result that some men connect with fertility, relationships, or masculine identity. Procedure-specific evidence indicates that collection itself may be burdensome: a fertility-clinic study documented discomfort with masturbation and occasional inability to produce a sample, men planning or attempting conception reported discomfort and difficulty arranging semen testing, and a randomized study found higher satisfaction with home than clinic collection [8-10]. Qualitative evidence on male participation in fertility research further shows that men may see fertility as mainly a women’s health issue, may question the relevance of their own participation, and may worry about semen-test results [11]. Social-science literature has also noted that men have been relatively marginalized in infertility research and psychosocial accounts [12].

Delayed or incomplete male evaluation may contribute to female-centered diagnostic and treatment pathways. U.S. national-survey and couple-level data indicate that male evaluation remains incomplete in a substantial minority of infertile couples and that inadequate specialist evaluation after abnormal semen findings may leave female partners carrying a disproportionate share of the care burden [13,14]. Improving semen-analysis acceptability is neither special accommodation for men nor a campaign for compliance; it may help promote more equitable couple care while preserving voluntary choice.

For this review, acceptability is treated as a service-process construct rather than as a synonym for uptake. It concerns whether the offer and testing process are understandable, personally relevant, emotionally safe, feasible, and voluntary; result interpretation and care connection are examined as downstream requirements of a clinically adequate service pathway when testing is chosen. The primary focus is adult men undergoing or considering semen analysis in infertility evaluation, male-infertility/urology care, or preconception/couple care, with other settings used only as secondary context. Existing evidence is dispersed across clinical, behavioral, psychosocial, and technological literatures and rarely distinguishes barriers before an informed decision, between a decision to test and sample submission, and after testing. Low uptake should therefore not be interpreted too quickly as informed refusal. Informed-choice and decision-quality literature distinguishes a decision reached with relevant knowledge and alignment with the person’s informed preferences from noncompletion caused by inadequate understanding or barriers that prevent an intended action [15-18]. Accordingly, this review examines which factors influence willingness, informed decision, and sample submission; where those factors may operate across the service sequence and settings; and what provisional service-design responses are suggested by the available evidence and clinical guidance. The nine-stage sequence does not replace or modify established acceptability or health-behavior frameworks; rather, it applies their constructs to potentially observable semen-analysis service events and is itself an author-developed, hypothesis-generating process map requiring prospective validation.

## Review

Review questions, scope, evidence selection, and narrative synthesis

This article is a targeted narrative review that includes an author-developed, provisional service-process map. It summarizes clinical, behavioral, psychosocial, technological, and Japanese-context evidence relevant to semen-analysis acceptability. It does not report new empirical data, reanalyze participant-level data from prior studies, or establish a new behavioral theory.

The primary population of interest was adult men undergoing or considering semen analysis in infertility evaluation, male-infertility/urology care, or preconception/couple care; partner perspectives were included when directly relevant to shared planning or care delivery. Evidence from general men’s health, voluntary fertility or reproductive-health research, and workplace-adjacent settings was included only when it informed a specific pathway stage and was interpreted as secondary, setting-specific evidence. The review was not intended to support population-wide semen screening.

Semen analysis primarily referred to conventional laboratory examination of an ejaculated semen sample. Home collection followed by validated laboratory analysis was included as an alternative collection setting. Mail-in systems, home-based test kits, rapid or smartphone tools, and point-of-care devices were considered only when they informed feasibility, access, result interpretation, or care linkage; their scope and validity were evaluated according to the specific approach and were not assumed to be equivalent to comprehensive laboratory semen analysis. Consistent with the operational definition above, awareness, willingness, informed decision, participation, completion through sample submission, collection satisfaction, result receipt and interpretation, and referral were treated as related but distinct service-process indicators rather than interchangeable measures of acceptability.

The review addressed three questions: (1) What individual, interpersonal, clinical, and service-level factors influence men’s willingness to undergo semen analysis, their informed decision, and subsequent sample submission? (2) At which stages, from information reach and comprehension through informed decision, sample submission, result interpretation, and care connection, do these factors operate, and how do they differ across settings? (3) What provisional service-design responses are suggested by the available evidence and clinical guidance for reducing avoidable barriers while preserving informed and voluntary choice, privacy, analytical validity, and appropriate clinical follow-up?

Literature searching was iterative, targeted, and question-driven rather than exhaustive. We searched PubMed/MEDLINE and used Google Scholar for citation chasing, together with CiNii Research, Japan Science and Technology Information Aggregator, Electronic (J-STAGE), and clinical guideline or society webpages. No lower publication-date limit was imposed because foundational frameworks and earlier seminal sources were eligible; searches and citation chasing were updated through August 7, 2026. The synthesis was limited to sources that the authors could evaluate in English or Japanese. Search domains and example concepts are shown in Table 1. Because search terms were refined according to the evidence domain and source, no single Boolean strategy generated the complete cited corpus.

**Table 1:** Search domains, example concepts, source types, and evidentiary functions in the narrative synthesis J-STAGE: Japan Science and Technology Information Aggregator, Electronic; TFA: Theoretical Framework of Acceptability; COM-B: capability, opportunity, motivation, and behavior The terms shown are examples of concepts used in iterative, domain-specific searches and do not represent a single uniform Boolean strategy applied across all sources. Evidence function was assigned at the level of the claim supported. A source was treated as direct only for the outcomes it examined and was not generalized to unmeasured stages or settings. “Provisional service-design implication” denotes an author-derived synthesis unless the statement is explicitly attributed to a cited guideline, laboratory standard, or intervention study. These labels do not constitute a formal certainty rating

Search domain	Example search concepts	Principal sources	Evidentiary function	Role in synthesis
Semen analysis and semen testing	semen analysis; semen testing; sperm test; male fertility testing; semen collection	PubMed/MEDLINE; Google Scholar; guideline webpages	Direct semen-analysis evidence; clinical or laboratory guidance	Clinical rationale; uptake barriers; collection feasibility; convenience safeguards
Male infertility and clinical care	Male infertility; male-factor infertility; urology referral; male reproductive health	PubMed/MEDLINE; guideline webpages; J-STAGE if relevant	Related clinical evidence; clinical guidelines	Clinical pathway; referral; result interpretation; Japanese clinical context
Acceptability and health behavior	Acceptability; TFA; COM-B; Health Belief Model; Theory of Planned Behavior; behavior change; informed choice; decision quality; shared decision making; patient decision aids; informed refusal	PubMed/MEDLINE; Google Scholar	Theoretical or framework evidence	Conceptual mapping; ethical distinction between pathway failure and legitimate refusal; generation of candidate measures for future operationalization and validation
Male participation, stigma, and masculinity	Male infertility stigma; masculinity; embarrassment; result anxiety; fertility research participation	PubMed/MEDLINE; Google Scholar; sociological databases as available	Related or indirect psychosocial evidence	Self-relevance; emotional safety; stigma; couple-care equity
Fertility literacy and preconception care	Fertility knowledge; men’s preconception health; fertility information; male fertility campaign	PubMed/MEDLINE; Google Scholar	Related or indirect evidence	Information reach; comprehension; self-relevance in preconception and couple care
Home testing and convenience	Home semen test; mail-in semen analysis; home collection; smartphone semen analysis; point-of-care semen analysis	PubMed/MEDLINE; Google Scholar	Direct or modality-specific semen-testing evidence	Collection feasibility; home testing; unsupported-endpoint safeguards
Japanese context	Japan male infertility; Japanese fertility knowledge; gender-role attitudes; infertility treatment and workplace privacy	PubMed/MEDLINE; CiNii; J-STAGE; selected policy sources	Contextual or indirect evidence; policy and guideline sources	Japanese implementation context; privacy; work-treatment balance; contextual safeguards

Evidence selection was question-driven, purposive, and iterative. Sources were retained when they addressed at least one of the three review questions and served a defined evidentiary function: direct empirical evidence on an offered semen-analysis procedure, sample collection or submission, modality-specific test performance, result return, or follow-up; related evidence from male-infertility, fertility-literacy, psychosocial, or Japanese-context research linked to a specified pathway stage; foundational theory or decision-science literature; or clinical, laboratory, and selected policy guidance. Sources were generally not retained when they lacked relevance to acceptability or service delivery, consisted primarily of commercial material without independent evidence, concerned adjacent settings with limited transferability, could not be evaluated by the authors in English or Japanese, or duplicated a more direct, current, or authoritative source. Candidate sources were identified through database searching, citation chasing, guideline and society websites, and claim-specific supplementary searches during drafting and revision. The authors reviewed the final cited sources and made the final decisions regarding source retention and claim linkage. These were general selection principles, not prospectively registered eligibility criteria; no independent duplicate screening or prospectively maintained screening log was used.

The cited evidence was synthesized narratively at the level of the claim. No standardized study-level data-extraction form was used. In interpreting each claim, the authors considered the source type, population and setting, outcome or construct examined, directness to semen analysis, and limits on transferability. Clinical and laboratory statements were anchored to relevant guidelines or standards; procedure-specific statements were supported by direct semen-analysis evidence where available; and indirect or contextual evidence was limited to the constructs and settings examined. Evidence was classified as direct semen-analysis evidence, related or indirect evidence, theory or framework evidence, clinical or laboratory guidance, or author-derived service-design inference. No meta-analysis, formal study-level risk-of-bias assessment, or certainty grading was performed. These evidence-function categories are not certainty grades; they were used to distinguish empirical findings from contextual extrapolation and provisional interpretation. The final cited evidence base comprises 60 sources. This count is not a record-screening denominator; the sources include empirical studies, reviews, frameworks, clinical guidelines, laboratory standards, and contextual documents.

The authors constructed the pathway by arranging recurring semen-analysis service events described across the reviewed literature in temporal order; the established constructs summarized in Table 2 were used to interpret and map onto, rather than empirically derive, those events. This was an interpretive author synthesis, not a prespecified coding exercise, independent dual-coding process, formal consensus method, external expert consultation, or empirical stage-identification procedure; stages were kept separate when they represented different service-design questions or candidate future endpoints and otherwise grouped for parsimony. The resulting nine-stage structure is therefore one provisional, hypothesis-generating organization rather than a uniquely correct taxonomy; its boundaries may overlap and require prospective operationalization and validation. Artificial intelligence (AI)-assisted tools supported the organization of candidate sources and preliminary evidence mapping during drafting; the authors made the final decisions regarding source retention, evidence-function classification, interpretation, and conclusions.

**Table 2:** Applying established acceptability and health-behavior frameworks to a provisional semen-analysis service sequence

Model or concept	Core construct or principle	Semen-analysis-specific translation	Analytic function in this review
Theoretical Framework of Acceptability [19]	Acceptability includes affective attitude, burden, perceived effectiveness, ethicality, intervention coherence, opportunity costs, and self-efficacy	Separates emotional safety, collection burden, test understanding, and confidence to complete the test	Defines what may be unacceptable without presenting the pathway as a new theory
COM-B and Behavior Change Wheel [20]	Behavior depends on capability, opportunity, and motivation	Knowledge, privacy, clinic hours, transport, collection options, and result anxiety can each block completion	Prevents uptake from being reduced to motivation alone
Theory of Planned Behavior [21]	Intention is shaped by attitudes, subjective norms, and perceived behavioral control; actual behavior may still diverge from intention when control is limited	Fertility-as-women’s-issue framing, partner expectations, and perceived control over collection may shape the decision or intention to test, whereas sample production and delivery constitute a separate behavioral endpoint	Distinguishes the decision or intention to test from subsequent sample submission, preserving an intention–behavior gap that can be measured prospectively
Health Belief Model [22]	Behavior depends on perceived susceptibility, benefits, and barriers	Confidence in fertility may lower perceived need; embarrassment, cost, and inconvenience may dominate perceived barriers	Explains self-relevance, perceived benefit, and perceived barriers
Process evaluation [23]	Reach, implementation, mechanisms, context, and participant response matter	A low completion rate should prompt stage-specific attrition analysis rather than a single refusal explanation	Supports analysis of where the service sequence fails
Informed choice, decision quality, shared decision making, and meaningful nonconsent [15-18]	A high-quality decision requires relevant knowledge, realistic expectations, explicit awareness that a choice exists, and deliberation that results in a decision aligned with the person’s goals and preferences	Before classifying non-uptake as refusal, assess whether the man understood the purpose, limits, options, and likely consequences of semen analysis, had a feasible opportunity to act, and made an informed decision to proceed, defer, or decline	Defines the ethical boundary between legitimate refusal or deferment and avoidable pathway failure, while keeping decision quality distinct from subsequent sample submission

Applying existing acceptability frameworks to a provisional semen-analysis service sequence

Acceptability is already well-developed in health services and behavior change research. The Theoretical Framework of Acceptability; Capability, Opportunity, Motivation, Behavior (COM-B); the Theory of Planned Behavior; the Health Belief Model; process-evaluation guidance; and informed-choice and shared decision-making literature provide constructs relevant to semen-analysis uptake [15-23]. These frameworks are primarily construct-based and are not semen-analysis-specific process maps: they help explain what may influence acceptability or behavior, but they do not locate whether attrition occurred before an informed decision, between a decision to test and laboratory receipt of a sample, or after testing during result interpretation and care connection. The proposed sequence therefore maps established constructs onto potentially observable semen-analysis service events to generate stage-specific hypotheses; it neither replaces the parent frameworks nor establishes empirically discrete stages or a validated model. Table 2 summarizes this conceptual application.

**Figure 1:**
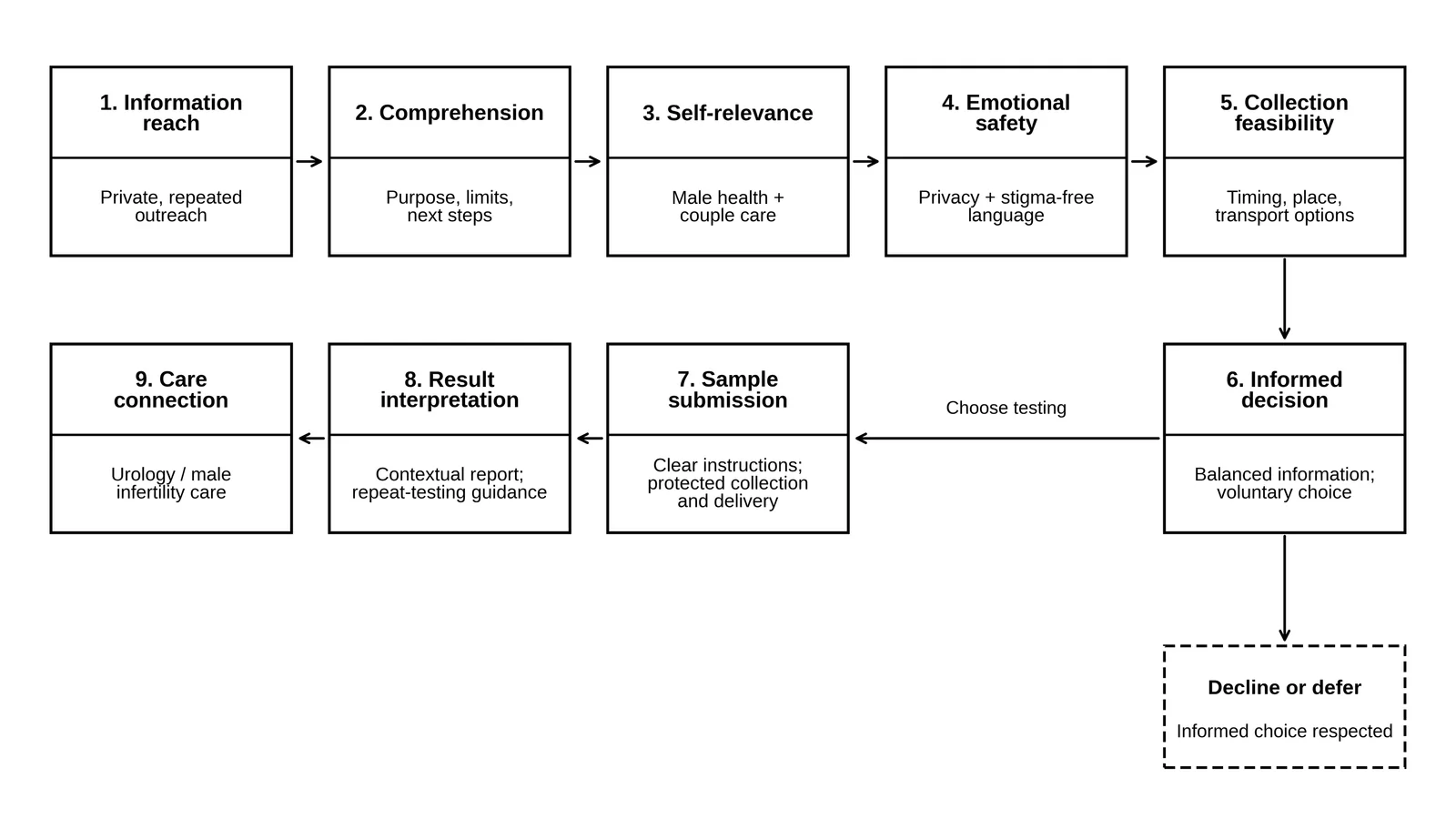
Provisional semen-analysis acceptability pathway as a service-design sequence The sequence extends from information reach to care connection. Each numbered box pairs a service event with a provisional service-design response for future evaluation. Informed decision is represented as a branching point: an informed decision to decline or defer is a legitimate outcome for the current testing offer, whereas those choosing testing may proceed to Stage 7, sample submission. Separating informed decision from sample submission preserves the distinction between intention and behavior and allows non-submission after a decision to test to be examined prospectively. The dashed box denotes a legitimate alternative outcome rather than pathway failure. Stage boundaries are conceptual, may overlap, and have not yet been empirically validated. The figure is an original conceptual synthesis and does not present new data or a model derived from the authors’ prior workplace-adjacent survey

Applied in this way, the provisional sequence comprises nine service events: information reach, comprehension, self-relevance, emotional safety, collection feasibility, informed decision, sample submission, result interpretation, and care connection. The informed-decision step is a branching point. Informed-choice and shared decision-making literature indicates that a high-quality decision requires relevant knowledge, realistic expectations, explicit recognition of the choice, and deliberation about goals and preferences [15-18]. A decision aligned with the person’s informed preferences, including a decision to decline or defer, is therefore a legitimate endpoint. Those choosing testing may proceed to sample submission, which remains a separate behavioral endpoint. Separating informed decision from sample submission preserves the intention-behavior distinction and allows nonsubmission after a decision to test to be examined prospectively. Figure 1 summarizes the sequence and corresponding service-design responses.

This translation is important because noncompletion is not always equivalent to informed refusal. A man who never receives the invitation, assumes that fertility is only his partner’s issue, fears the implications of an abnormal result, or cannot arrange collection during working hours has not reached the same decision point as a man who receives clear information, feasible options, and supportive result-return arrangements [15]. Direct and related evidence on semen testing and male fertility participation supports the same stage-based interpretation: reported barriers include confidence in fertility, cost, discomfort, inconvenience, stigma, fertility being framed as women’s health, uncertainty about participation value, and concern about semen-test results [9,11].

The main bottleneck may differ by setting. In infertility care, clinical relevance may already be clear, so emotional safety, collection logistics, and result interpretation may dominate. In preconception or couple care, information reach and self-relevance may be earlier barriers. In voluntary research, the distinction between meaningful refusal and failure to reach an informed decision point is especially important. Thus, the sequence is intended to generate testable, setting-specific hypotheses about where attrition occurs and which responses warrant evaluation, rather than to establish a competing theory, empirically discrete stages, or a validated intervention model.

Attitudes toward semen analysis and barriers to uptake

If the acceptability pathway is a service sequence rather than a new theory, the next question is where the sequence may break down. Direct and related evidence indicates that nonuptake should not be collapsed into one category of refusal. Men may not see semen analysis as relevant, may anticipate stigma or an unwanted result, may find collection difficult, or may never reach an informed decision point [9-11,15,24]. Table 3 summarizes major barrier patterns, representative evidence anchors, and stage-specific service-design responses.

**Table 3:** Barrier domains, evidence functions, and provisional stage-specific service-design implications Unless explicitly attributed to clinical guidance, a laboratory standard, or direct intervention evidence, the service-design implications in Tables 3–7 are author-derived proposals for future evaluation rather than established intervention effects

Pathway domain	Barrier pattern	Provisional service-design implication	Evidence function and representative references
Self-relevance	Confidence in fertility, uncertainty about participation value, fertility framed as women’s health, and limited male invitation can make semen analysis feel irrelevant	Explain male reproductive health and couple-care relevance without moral obligation	Mixed direct and related/indirect evidence: [9,11,24]
Emotional safety and result anxiety	Fear of results, discomfort performing the test, perceived stigma, semen-result concern, and identity-linked interpretation can interrupt uptake	Use stigma-free language, privacy protection, and anticipatory explanation of result meaning	Mixed direct and related/indirect evidence: [9,11,25-28]
Collection feasibility	Anticipated timing, privacy, transport, cost, discomfort with masturbation-based collection, and other collection conditions may make testing appear impracticable before a decision is reached	Reduce anticipated burden and offer analytically valid collection and submission options	Direct semen-analysis evidence: [8-10]
Informed decision	Observed non-uptake may not represent a high-quality decision when the choice is not explicit, knowledge or expectations are inadequate, or the decision does not reflect the person’s goals and preferences	Make the choice explicit; explain reasonable options and consequences; elicit goals and preferences; support deliberation; and respect an informed decision to proceed, defer, or decline	Theoretical/framework and decision-science evidence: [15-18]
Sample submission	A decision or stated intention to test may not lead to completed collection and laboratory receipt of a sample	Provide clear instructions, privacy-protected collection, and feasible delivery arrangements; measure non-submission separately and assess its reasons	Direct and theoretical/framework evidence: [9,10,21]
Setting interaction	Infertility care may have high clinical relevance but ongoing emotional and logistical barriers; preconception care may have upstream relevance and literacy barriers	Identify the likely bottleneck by setting rather than applying a single uptake explanation	Mixed evidence and author-derived cross-setting inference: [9-11,24]

The subsequent informed-decision stage is distinct from the behavioral endpoint of sample submission. A decision to proceed, defer, or decline should follow explicit recognition that a choice exists, adequate understanding of the test and its consequences, and deliberation about goals and preferences [15-18]. A man may choose testing without a sample subsequently being received by the laboratory [21]. An informed decision to decline or defer should be respected, whereas nonsubmission among those choosing testing should be recorded and interpreted separately rather than retrospectively classified as refusal.

These barriers interact. A man in preconception care may have low self-relevance, whereas a man in infertility care may already accept the clinical rationale but still encounter collection or result-related barriers. Accordingly, nonuptake should be classified according to whether the informed-decision stage was reached and, if testing was chosen, whether sample submission occurred, rather than treated as a unitary refusal outcome.

Result anxiety, masculine identity, and difficulty talking about fertility

Fear of results, stigma, and discomfort should not be interpreted as evidence that men are irrational, irresponsible, or unwilling to participate in fertility care. These barriers are better understood as emotional-safety barriers. Semen analysis asks men to provide a private sexual specimen and to anticipate a result that may carry personal and social meaning beyond its clinical interpretation.

Direct semen-testing evidence supports this view. Reported barriers include fear of results, discomfort performing the test, and perceived stigma [9]. Qualitative evidence on fertility research participation also suggests that semen-test results can cause concern. It also shows that fertility can be framed mainly as a women’s health issue, which makes male involvement less expected or less openly discussed [11]. Result anxiety can therefore begin before the test is performed. A man may avoid testing not because he has made a firm decision against male evaluation, but because he imagines what an abnormal result might mean for himself, his partner, or how others see him.

This anticipated burden is intensified by the social meaning of male-factor infertility. Hanna and Gough’s qualitative questionnaire study shows that male infertility can be socially constructed through silence, stigma, and marginalization in reproductive medicine and wider social life [25]. Dolan and colleagues also describe men’s experiences of infertility as involving threats to masculine identity, although the meaning and intensity of this threat differ across men and contexts [26]. The practical point is that an offer of semen analysis can be heard through these social meanings, even when the clinician intends it as a routine diagnostic step.

Public language can strengthen this threat. Gannon et al. showed that male infertility can be represented through masculine stereotypes and, in some contexts, confused with sexual dysfunction [27]. Such framing can make a routine clinical test feel like a judgment of the person rather than a source of clinical information, so emotional safety may fail before collection occurs.

This difficulty is not limited to men who already have an infertility diagnosis. Work on men’s talk about fertility suggests that fertility may not become an explicit personal issue before it is recognized as a problem [28]. This helps explain why semen analysis can feel sudden or personally exposing in preconception and early conception-planning contexts. If fertility has not previously been framed as part of men’s health or couple care, the first invitation to produce a semen sample may carry more symbolic weight than clinicians or researchers expect.

One provisional service-design implication is to acknowledge this burden and use clear, nonjudgmental communication rather than appeal to masculinity. Clinical and research communication should say plainly that semen analysis is a reproductive-health test, not a measure of sexual function or personal worth. Communication should normalize possible anxiety, protect privacy, and explain how results will be returned and what support will follow. These emotional-safety barriers and corresponding service-design responses are summarized in Table 3.

Information reach, fertility literacy, and self-relevance

Information about male fertility may not reach men in a form that is understandable, relevant, and usable. In the acceptability pathway, information reach and comprehension are conditions for self-relevance, not minor preliminaries. A man cannot make a meaningful decision about semen analysis if he has not been addressed as a reproductive-health subject or does not understand why male-factor assessment may matter for himself or the couple. Table 4 summarizes evidence relevant to information reach, fertility literacy, and self-relevance.

**Table 4:** Related or indirect evidence for information reach, fertility literacy, and self-relevance, with provisional pathway implications The evidence summarized in this table is related or indirect with respect to semen-analysis acceptability unless otherwise specified. Its use is limited to the pathway construct and setting examined

Evidence stream	Evidence summary	Pathway implication	Provisional service-design implication
Men’s fertility knowledge [29]	Population-based evidence indicates limited awareness of factors associated with male infertility	Information reach and comprehension cannot be assumed	Explain male fertility as part of reproductive health before crisis
Preconception-health knowledge [30]	Systematic-review evidence suggests men’s preconception-health knowledge is generally low and understudied	Semen analysis may not seem relevant until a pathway makes it relevant	Use preconception and couple-care encounters to make male-factor assessment understandable
Men’s talk about fertility [28]	Fertility may not become a personal issue before it is recognized as a problem	Awareness does not automatically become self-relevance	Connect information to life stage, couple planning, and non-judgmental next steps
Fertility-information preferences [31]	Young men preferred accurate, reliable, understandable information and criticized masculinity-linked messaging	Male-targeted communication can fail if poorly timed, public, directive, or identity-linked	Use private, reliable channels and avoid masculinity imagery or shame-based appeals
Education interventions [32]	Interventions can improve male fertility knowledge, but design and evidence quality matter	Knowledge improvement should support informed choice, not pressure to test	Pair education with privacy, test limitations, repeat-testing guidance, and referral visibility

The evidence supports treating fertility literacy as a real upstream barrier. Men’s fertility knowledge and preconception-health knowledge are often limited; fertility may not become personally salient until it is recognized as a problem, and male-targeted fertility information may fail when it is poorly timed, unclear, too public, too directive, or linked to masculinity [28-31]. Intervention evidence suggests that male fertility knowledge can be improved, but awareness alone is not enough unless communication also reduces avoidable shame, clarifies privacy, and makes next steps visible [32].

These findings suggest that information may need to be staged according to context. General education can normalize male fertility as part of reproductive health. Preconception and couple-care encounters can connect that knowledge to shared reproductive planning without pressure. Infertility care can focus on what semen analysis measures, what it does not measure, why repeat testing may be needed, and how results will be interpreted and connected to care. The goal is not to convert every man into a test recipient, but to make the decision point informed, private, nonshaming, and clinically meaningful, as summarized in Table 4.

Not special treatment for men, but equity in couple care

Male-focused communication may seem to be a call for extra accommodation for men. That interpretation should be avoided. The purpose of improving semen-analysis acceptability is not to make men a privileged group within fertility care. Rather, making male evaluation more reachable and clinically connected may help reduce avoidable female-centered diagnostic and treatment burden and support a more balanced couple-care pathway [13,14].

A fair approach to male engagement is also consistent with medical-anthropological work showing that men can influence women’s reproductive health. This work argues that reproductive-health programs should consider how men can be included fairly, rather than whether they should be included at all [33]. Applied to semen analysis, this supports a partner-inclusive but noncoercive pathway. Male evaluation should become visible, understandable, and clinically connected within couple care, while individual confidentiality is preserved and partner pressure is avoided.

This equity frame is necessary because infertility care has long been shaped by gendered expectations. Global infertility scholarship has described how infertility, reproductive technologies, and treatment pathways are organized through unequal social and clinical expectations placed on women and men [34]. Social-science literature has also noted the relative marginalization of men in infertility research and psychosocial accounts. This can make male infertility and male evaluation less visible [12]. In this context, asking whether semen analysis is acceptable is not only a question of patient convenience. It is part of whether male-factor assessment becomes visible, timely, and clinically connected within couple care.

The clinical rationale is also important. Japanese male infertility guidance places semen evaluation within a broader urological pathway [1]. The current AUA/ASRM guideline, as amended in 2024, and the 2025 European Association of Urology (EAU) update both place male assessment within infertile-couple evaluation. The AUA/ASRM guideline further recommends evaluation by a male reproductive expert when one or more semen parameters are abnormal or male infertility is presumed [2,3,35]. If semen analysis is not offered, understood, completed, interpreted, or connected to care, male-factor evaluation may be delayed while female partners continue through more visible diagnostic and treatment pathways [13,14].

The barrier is not simply that men are absent from care. Men may not always be clearly invited into reproductive-health conversations. In qualitative work on male participation in fertility research, men described fertility as being framed as a women’s health issue and were sometimes uncertain about the relevance or value of their own involvement [11]. Grace et al. also reported that men may not recognize that they are being invited into fertility and reproductive-health discussions [36]. These findings support the argument that information reach and self-relevance depend partly on how fertility care addresses, or fails to address, men as reproductive-health participants.

In couple care, one proposed approach is to address both partners in the initial explanation while maintaining a private route for the man’s questions, scheduling, collection instructions, and result discussion. Partner inclusion does not remove the need for individual dignity and confidentiality. The result-return plan should be explained before testing, so semen analysis is not seen as an isolated judgment but as one step in a pathway that may include repeat testing or referral.

At the same time, couple-care equity cannot justify coercion. Partner-inclusive communication should preserve explicit choice, balanced information, and deliberation about the man’s goals and preferences; an informed decision to decline or defer after that process should be respected [15-18]. The clinical task is to reduce avoidable barriers to choice, not to replace one form of gendered burden with another form of pressure. Setting-specific implications and safeguards are summarized in Table 5. Infertility care, male-infertility/urology care, and preconception/couple care constitute the primary scope, whereas general men’s health and voluntary research are included as secondary contexts.

**Table 5:** Author-derived setting-level synthesis of provisional implications and safeguards for semen-analysis acceptability Table 5 is an author-derived cross-setting synthesis based on the evidence cited in Tables 3 and 4 and the accompanying text; references are therefore not repeated

Setting	Likely bottleneck	Provisional service-design implication	Safeguard
Infertility care	Emotional safety, collection feasibility, result interpretation, referral	Assign a named ordering or fertility-clinic clinician to communicate the result, arrange repeat conventional semen analysis when indicated, and initiate and track specialist referral while integrating the female partner’s evaluation and treatment timeline	Respect informed refusal and privacy; do not allow repeat testing to delay specialist evaluation of severe or clinically concerning findings
Male-infertility/urology care	Fragmented pathway from result to male evaluation	Perform etiologic history and examination, direct endocrine, genetic, or imaging evaluation when indicated, provide management, and communicate the plan back to the fertility clinic	Avoid isolated result disclosure, untracked referral, or loss of couple-level care coordination
Preconception/couple care	Low self-relevance and concern about overmedicalization	Provide optional, context-sensitive information about male fertility and semen analysis when relevant	Avoid population-screening rhetoric, pronatalist pressure, and masculinity-based appeals
General men’s health	Low fertility literacy and low perceived relevance	Include male reproductive health as one possible part of broader men’s health conversations	Do not treat semen analysis as a stand-alone general-health screening test
Voluntary fertility/reproductive-health research	Unclear benefit, consent complexity, result-return uncertainty	State purpose, burden, privacy protections, result-return options, and referral resources before participation	Separate research invitation from clinical indication
Primary care or first-contact clinical care	Male-factor concerns, general-health contributors, or referral red flags are not recognized, and initial evaluation or referral is fragmented	Obtain or coordinate reproductive and medical histories; review medications, lifestyle factors, and comorbidities; perform a focused examination where appropriate; arrange baseline conventional semen analysis and indicated hormonal testing; communicate the interim plan; and initiate and track specialist referral	Primary care should streamline, not replace, specialist etiologic evaluation. Repeat testing should not postpone referral for severe or otherwise clinically concerning findings

Convenience, home testing, and the risk of unsupported endpoints

A pathway designed for equitable couple care must be easier to complete, but easier testing is not automatically better care. Home collection, home-based tests, smartphone or point-of-care tools, rapid tests, and flexible submission can reduce embarrassment, travel, timing, and privacy barriers. Yet they can become unsupported endpoints if results are returned without interpretation, repeat-testing guidance, or access to male reproductive care.

The literature supports a balanced approach. Home collection can improve satisfaction in selected infertility settings without necessarily changing conventional semen parameters when collection and transport requirements are met [10]. Reviews of home male-fertility testing and emerging home-based semen-analysis technologies describe tools that may improve privacy, convenience, and access, and smartphone or paper-based point-of-care approaches illustrate how testing may move closer to the patient [37-39]. These approaches are relevant to acceptability because they may help some men enter a pathway that would otherwise be avoided.

Convenience alone is not sufficient. Home or rapid tests may measure only selected semen features [37,40], and even conventional semen analysis has limits as a test of fertility [41]. Accuracy is device-, endpoint-, and protocol-specific: a smartphone-based home test was evaluated against a laboratory analyzer for motile sperm concentration rather than for all components of comprehensive semen analysis [42], while mail-in analysis may be affected by transport and delayed assessment, particularly for motility and morphology [43]. Standardized preanalytical, analytical, and postanalytical procedures with quality control and quality assurance remain necessary [5,44]. A simplified reassuring result may delay evaluation when symptoms, history, or couple infertility warrant assessment; an abnormal, borderline, invalid, or inconclusive result may create anxiety without context. Clinically meaningful pathways should therefore specify what the test measures, what it does not measure, when validated conventional laboratory testing or repeat testing is needed, how results will be explained, who is responsible for follow-up, and where care is available. For simplified home tests that are not equivalent to comprehensive laboratory analysis, abnormal, borderline, invalid, or inconclusive results should lead to validated conventional laboratory semen analysis; a reassuring result should not close the evaluation when infertility or another clinical concern persists [37,40].

After an initially abnormal conventional semen analysis, or a borderline or inconclusive result when clinical uncertainty remains, repeat conventional semen analysis should be considered under standardized conditions. Because semen parameters are biologically variable, the current AUA/ASRM guideline, amended in 2024, notes that at least two semen analyses obtained approximately one month apart are important to consider, especially when the first analysis is abnormal; the 2025 EAU update similarly recommends at least two consecutive analyses after an abnormal baseline result [3,35,45]. The interval may be individualized according to the initial finding and clinical context. Repeat testing should not delay specialist evaluation when the initial finding is severe or otherwise clinically concerning; in such cases, repeat analysis and referral may proceed in parallel.

Consistent with the amended AUA/ASRM guideline, men with one or more abnormal semen parameters or presumed male infertility should be evaluated by a male reproductive expert. The amended guideline also recommends evaluation of the male partner after failed assisted reproductive technology (ART) cycles or recurrent pregnancy losses involving two or more losses [3]. Findings that may warrant prompt or parallel referral include azoospermia, severe oligozoospermia, multiple semen abnormalities, complete asthenozoospermia or necrozoospermia, persistent low semen volume suggesting obstruction or ejaculatory dysfunction, and an abnormal reproductive history or physical examination [35,46]. Increased round cells or pyospermia require directed evaluation to distinguish leukocytes from germ cells and to assess for infection when appropriate [3].

Responsibility should be explicit and closed-loop. As a pragmatic service-design allocation, a named clinician, usually the ordering clinician in the fertility clinic or primary-care setting, should communicate and contextualize the result, explain biological variability and the limitations of a single value, arrange repeat testing, and initiate and track referral rather than relying on the laboratory report alone [47,48]. The andrology laboratory should ensure specimen adequacy, validated analysis, quality control and assurance, and clear identification of invalid or technically limited specimens [44]. The fertility clinic should integrate the male findings with the female partner’s evaluation and treatment timeline; primary care may initiate or coordinate the baseline male-factor work-up; and the male reproductive expert should direct etiologic evaluation and indicated management [35,48,49].

The current AUA/ASRM guideline further states that semen-analysis results should guide management and that findings are generally of greatest clinical significance when multiple abnormalities are present [3]. Together with Japanese and EAU guidance, these recommendations support connecting abnormal or persistent findings to etiologic evaluation and, when indicated, endocrine, genetic, imaging, medical, surgical, or assisted-reproductive management rather than treating results as isolated numbers [1-3,35,50]. Result-return guidance, particularly relevant to research and nontraditional testing pathways, similarly emphasizes validity, interpretation, value, participant understanding, and support [51]. The corresponding division of responsibilities is summarized by setting in Table 5. Table 6 summarizes convenience-oriented approaches and the safeguards required to keep testing connected to interpretation and care. Thus, convenience should be treated as a pathway modifier rather than an endpoint (Table 6).

**Table 6:** Convenience-oriented approaches and safeguards against unsupported endpoints ART: assisted reproductive technology

Convenience-oriented approach	Barrier addressed	Safeguard required for care connection	Representative references
Home collection	Clinic-based masturbation, embarrassment, time pressure, and privacy burden	Specify abstinence period, container, temperature, transport time, and laboratory requirements	[10]
Flexible drop-off or mail-in submission	Work schedule, travel, transport, and privacy burden	Use only where validated; avoid compromising sample quality	[37,38]
Home-based male fertility testing	Privacy and access barriers	For simplified tests that are not equivalent to comprehensive laboratory analysis, abnormal, borderline, invalid, or inconclusive results should lead to validated conventional laboratory semen analysis. A reassuring result should not close the evaluation when infertility or another clinical concern persists	[37,38,40]
Smartphone or point-of-care tools	Clinic access and rapid-feedback barriers	Require validation, clear limitations, confirmatory testing, and clinical interpretation	[39]
Contextualized result reports	Result avoidance, confusion, anxiety, or overinterpretation	Assign a named clinician to explain biological variability, technical and interpretive limitations, the repeat-testing plan, and the referral plan. The laboratory should clearly identify invalid, incomplete, or technically limited specimens	[41,44,47,48,51]
Referral and management pathway	Attrition after abnormal findings or repeated reproductive failure	Arrange evaluation by a male reproductive expert for one or more abnormal conventional semen parameters or presumed male infertility. The male partner should also be evaluated after failed ART cycles or recurrent pregnancy losses involving two or more losses. Repeat testing and referral should proceed in parallel when the initial finding is severe or otherwise clinically concerning. Use the semen-analysis findings, particularly the presence of multiple abnormalities, to guide subsequent evaluation and management, and confirm completion of the clinical handoff	[1-3,35,46,47,50]

Japanese context: male infertility, sociological silence, work-treatment balance, and noncoercive reproductive policy

The safeguard principle described above has a specific meaning in Japan. The question is not only whether semen analysis is available, but whether clinical, informational, and social pathways make male evaluation understandable, private, clinically connected, and compatible with ordinary work and couple life. Japanese male infertility guidance provides a urological pathway for semen evaluation, but guideline availability does not by itself solve information reach, result anxiety, collection timing, or follow-up [1]. Table 7 summarizes Japanese contextual issues, their acceptability implications, and privacy-preserving practice responses.

**Table 7:** Contextual Japanese evidence and provisional implications for privacy-preserving couple care

Japanese contextual issue	Acceptability implication	Provisional practice implication	Representative references
Male infertility clinical pathway	Semen analysis belongs in male infertility or urological care, but guideline availability does not guarantee uptake	Link semen results to repeat testing, interpretation, and urology or male infertility care	[1]
Sociological silence and embodied male infertility	Semen analysis may expose a private bodily or identity-linked condition	Use privacy-protective, non-judgmental language when offering testing and returning results	[52]
Fertility-knowledge gaps in Japan	Male fertility and semen analysis cannot be assumed to be self-evident or personally relevant	Provide plain-language fertility and semen-analysis information in preconception or couple-care settings	[53]
Gender-role attitudes and fertility intentions	Reproductive planning is shaped by gendered expectations	Frame semen analysis as shared reproductive planning without partner pressure or male-compliance messaging	[54]
Work-treatment balance and workplace-adjacent feasibility	National survey evidence indicates that employer support for infertility treatment is not universal; workplace-adjacent semen-analysis contexts may additionally involve information-reach, collection, result-anxiety, privacy, and disclosure barriers	Use flexible scheduling and privacy-preserving support; maintain voluntary participation and do not require disclosure of semen analysis or diagnosis	[55-57]
Low-fertility and reproductive-policy context	Low fertility makes reproductive-health support socially salient, but policy and scholarly sources emphasize self-decision, voluntariness, and the risk that reproductive support may be interpreted as social or policy pressure	Do not frame semen analysis as a demographic intervention or as reproductive duty; provide information only when clinically or personally relevant, using voluntary, privacy-preserving, non-pronatalist communication	[58-60]

Japan’s low-fertility policy context is therefore treated here as a contextual safeguard rather than as a reason to encourage semen analysis. The official English explanatory material for the General Principles for Child-Related Measures emphasizes individual hopes, self-selection, and self-decision [58], while scholarship on Japan’s ART context warns that normalized reproductive technologies may be shaped by societal, governmental, and industry pressures [59]. A public-health commentary on Japan’s birthrate policy similarly notes the tension between encouraging marriage and childbirth and insufficient spontaneity [60]. Applied to semen analysis, these sources support offering information only when clinically or personally relevant, using voluntary, privacy-preserving, nonpronatalist communication rather than framing testing as a demographic intervention or reproductive duty.

Japanese sociological work on azoospermia indicates that male infertility can be an embodied and private experience, not merely a reproductive label [52]. Japanese fertility-knowledge research also shows that fertility literacy cannot be assumed [53]. Gender-role evidence suggests that reproductive intentions and planning are embedded in gendered expectations [54]. Together, these sources support a Japanese pathway that frames semen analysis as shared reproductive planning and male reproductive health through privacy-protective, nonjudgmental communication.

Work-treatment balance is a practical part of the same pathway. Semen analysis may require abstinence timing, clinic or laboratory hours, sample transport, and time away from work. In the official FY2023 survey commissioned by the Japanese Ministry of Health, Labour and Welfare, 26.5% of responding companies reported either formalized support systems or case-by-case measures for employees undergoing infertility treatment [55,56]. Our group’s previously published formative workplace-adjacent follow-up survey [57] examined several acceptability-related components of an offered semen-analysis protocol, including information reach, low perceived relevance, resistance to semen collection, result anxiety, and privacy concerns. Although the setting differs from clinical infertility care and the study does not validate the pathway, its findings provide a setting-specific example of why uptake alone cannot establish whether a high-quality informed decision was reached [15-18,57]. This synthesis therefore proposes privacy-preserving communication, feasible scheduling, voluntary participation, and no required disclosure of participation or results to employers, as summarized in Table 7.

Limitations and future directions

This review has several limitations. First, it is a targeted narrative review, not a systematic review or meta-analysis. Literature searching and source selection were iterative and purposive rather than exhaustive or protocol-driven. Because source identification was not based on a prospectively maintained, deduplicated record set, a valid record-screening denominator was unavailable and was not retrospectively reconstructed. No Preferred Reporting Items for Systematic Reviews and Meta-analyses (PRISMA)-style flow, independent duplicate screening, formal study-level risk-of-bias appraisal, or certainty grading was undertaken. Although the search domains, general selection principles, and claim-level evidence functions are reported to make the synthesis more transparent and auditable, another review team might identify a different evidence set. The findings should therefore be interpreted as a claim-linked narrative synthesis rather than as a comprehensive or certainty-graded assessment of all eligible literature.

Second, direct evidence on semen-analysis acceptability is more limited than the broader literature on male infertility, masculinity, fertility literacy, and home testing. Some stages are supported by direct semen-analysis evidence, whereas others rely on related or indirect evidence, theoretical mapping, clinical guidance, or author-derived service-design inference. Accordingly, the proposed stage assignments and nonguideline service-design implications should be treated as hypotheses for prospective evaluation rather than as validated effects or graded recommendations. Third, settings differ substantially, and barriers in infertility care, preconception or couple care, voluntary research, and workplace-adjacent contexts are not identical. The nine-stage sequence is an author-developed organizing heuristic rather than an empirically segmented or validated model. Its stage count reflects the level of granularity selected for this review, not a uniquely correct or empirically optimal taxonomy; adjacent boundaries may overlap. Our group’s previously published formative workplace-adjacent follow-up survey [57] is cited only as a setting-specific illustration; no participant-level data from that study were reanalyzed, and the study neither generated nor validated the proposed pathway.

Future studies should prospectively define and measure the stages rather than reporting only final uptake or completion. Candidate endpoints include documented delivery and self-reported receipt of information, comprehension of the test’s purpose and limitations, patient-reported self-relevance and emotional safety, perceived collection feasibility, a recorded decision to proceed, defer, or decline, laboratory receipt of a sample among those choosing testing, documented result discussion, and completed referral when indicated. Within the informed-decision endpoint, decision quality could be assessed through decision-specific knowledge, realistic expectations about the test and its consequences, explicit awareness that a choice exists, clarity about goals and preferences, and concordance between those preferences and the recorded decision [16-18]. These decision-quality measures should be assessed before and separately from sample-submission behavior, so that an informed decision to decline or defer is recognized as a legitimate endpoint, whereas nonsubmission after choosing testing remains a separate service-process outcome. Future research should test whether adjacent stages are empirically distinguishable, whether the proposed measures are reliable and valid, and whether stage-specific interventions improve informed and voluntary completion without coercion.

## Conclusions

Across the settings reviewed, acceptability should not be inferred from uptake alone: informational, psychosocial, and practical barriers may arise at different stages and require stage- and setting-specific interpretation and service design rather than a single explanation of refusal. Semen analysis should be presented neither as a test of masculinity nor as a moral duty. It should be presented as a medical test that can support male reproductive evaluation, shared reproductive planning, and timely referral when clinically or personally relevant. Improving acceptability means designing pathways in which men can make informed, voluntary, unashamed, and clinically connected decisions within equitable couple care.
